# Exaggeration of Danger of Syphilitic Infection

**Published:** 1916-04

**Authors:** 


**Exaggeration of Danger of Syphilitic Infection.** Blaisdell, Boston M. & S. Jour. presents the following statistics: 60 known syphilitics exposed 1,227 individuals, 134 by intercourse, 442 by domestic relations, 651 by association at work. 5 of the 1,227 became infected, 1 a child by his mother's kiss, 4 by intercourse. While there is something to be said as to the possibility of infection by accidental contact, more or less indirect, it is significant that no case could be traced to association at work.

